# Consultations With Muslims From Minoritised Ethnic Communities Living in Deprived Areas: Identifying Inequities in Mental Health Care and Support

**DOI:** 10.1111/hex.14132

**Published:** 2024-07-02

**Authors:** Ashraf Tannerah, Oluwalolami Hazel, Sheree Desson, Rahima Farah, Zalihe Kamil‐Thomas, Halima Iqbal, Catrin Eames, Pooja Saini, Oladayo Bifarin

**Affiliations:** ^1^ School of Nursing and Advanced Practice, School of Psychology Liverpool John Moores University Liverpool UK; ^2^ Alder Hey Children's NHS Foundation Trust Liverpool UK; ^3^ Expert by Lived Experience; ^4^ Mersey Care NHS Foundation Trust Liverpool UK; ^5^ Central Liverpool Primary Care Network Liverpool UK; ^6^ Liverpool City Council Liverpool UK; ^7^ School of Nursing and Healthcare Leadership University of Bradford Bradford UK; ^8^ Senior Research Leader Programme National Institute for Health and Care Research (NIHR) London UK

**Keywords:** equity, mental health care, mental health support, Muslim

## Abstract

**Background:**

Limited research concerning existing inequities in mental health care and support services in the United Kingdom captures perceptions and lived experiences of the significantly underrepresented Muslim population.

**Methods:**

Underpinned by social constructivist theory, we used consultation to facilitate public and patient involvement and engagement (PPIE) to identify inequities in mental health care and support experienced by Muslims from minoritised ethnic communities living in deprived areas in Liverpool, UK. The rationale was to (a) better inform standards and policies in healthcare and (b) provide a psychologically safe space to members of the Muslim community to share perceptions and experiences of mental health care and support services. To ensure trustworthiness of the data, member checking was adopted. This paper describes the procedure to achieving this consultation, including our recruitment strategy, data collection and analysis as well as key findings.

**Findings:**

Twenty‐seven consultees attended the women's consultation and eight consultees attended the men's consultation. Consultees were from Yemeni, Somali, Sudanese, Egyptian, Algerian, Pakistani and Moroccan communities and share the Islamic faith. Four key interlinked themes were identified from consultees' narratives: (1) broken cycle of trust; (2) an overmedicalised model of care; (3) community mental health prevention initiatives; and (4) culturally conscious training and education.

**Conclusions:**

The Muslim population has identified numerous barriers to accessing mental health support and there is a need to resource activities that would aid deeper understanding of mental health support needs through continuous and meaningful community initiatives. This would afford mental health practitioners and organisations opportunities for developing realistic anti‐racism strategies, effectively adopting social prescription, strengthening partnerships and collaborations aimed at supporting delivery of evidence‐based mental health care provisions to tackle mental health inequities.

**Patient and Public Involvement:**

This paper reports on the involvement and engagement of Muslims from minoritised ethnic communities living in the Liverpool city region.

## 1 Background

The Muslim population in Liverpool, UK, comprises 25,763 people, marking a 2% increase from 2011 [1], and they face distinct challenges exacerbated by racism, Islamophobia and lack of culturally sensitive care [2, 3]. Intertwined with their social, religious and cultural identities are challenges which complicate engagement with mental health services [4]. Despite this, addressing mental health difficulties remains a universal challenge, influenced by a myriad of socioeconomic and cultural factors [5]. For instance, the World Health Organisation highlighted a significant treatment gap, with 75%–95% of individuals with severe mental health issues in low‐ to middle‐income countries, and 35%–50% in high‐income countries are not receiving necessary care [6]. As a high‐income country, the United Kingdom is not immune to these challenges, and the ongoing cost‐of‐living crisis is likely to exacerbate issues relating to absolute poverty. This is particularly true for individuals in households of Pakistani or Bangladeshi descent, who experience higher rates of relative low income [7]. Consequently, Muslims with lower socioeconomic status suffer disproportionately poor mental health outcomes [8, 9] and are further aggravated by higher health inequalities when compared to non‐Muslim communities [10]. Existing studies suggest that Muslims are less likely to seek treatment for mental health issues, a disparity linked to stigma, discrimination, cultural beliefs and financial constraints [9, 11, 12].

Over the past decade, the United Kingdom has increasingly recognised mental health care access as a policy priority [13], and yet, efforts to integrate social inclusion into research have been limited [14]. Existing care models often overlook the lived experience of ethnic minority communities, undermining the ethos of person‐centred care [15]. Moreover, recovery rates among Muslims are lower than those in other religious groups in the United Kingdom, indicating a pressing need for targeted interventions that account for the social and political dimensions of mental health in these communities [16, 17]. Addressing mental health needs of marginalised groups, including ethnic minorities, requires acknowledging their heightened vulnerability to poor outcomes and safety concerns within the healthcare system. These groups often seek formal healthcare as a last resort and face delays in diagnosis and care for mental health issues [18, 19, 20, 21]. Religious services frequently act as an initial support channel [22], with faith leaders playing a pivotal role in destigmatising mental health, offering counselling and facilitating referrals to mainstream services. This highlights the potential of collaborative efforts to enhance mental health outcomes for British Muslims [23].

Existing treatment gap in mental health treatment for Muslim communities in the United Kingdom remains a critical, multifaceted challenge necessitating a culturally sensitive, collaborative approach among healthcare providers, community leaders and policymakers to ensure equitable care access. Early intervention and prevention are crucial to mitigate the economic burden on the National Health Service (NHS) posed by crisis presentations among marginalised ethnic groups [24]. The role of religion as a coping mechanism is acknowledged within Islamic teachings, which recognise the significance of mental health and well‐being [25, 26, 27]. Despite this, societal perceptions often interpret mental health issues as tests from God, associated with shame [5]. Addressing the barriers to mental health support for Muslims, often described as ‘hard to reach’ [28] due to factors like language barriers and service accessibility, requires comprehensive, time‐intensive solutions and a variety of support options to meet diverse needs [29, 30].

The implementation of Integrated Care Systems (ICS) in England represents a new strategy for coordinating services, improving health and reducing inequalities [31]. Involving public advisors as equal partners in health and social care service research offers a promising avenue for addressing unmet health needs [32], especially in socio‐economically deprived areas like Liverpool. This paper outlines the recruitment of minoritised ethnic communities in Liverpool for consultations, the conduct of these consultations and the significant findings and implications for research and practice. It also discusses the strengths and limitation of consultation as a method, aimed at deepening our understanding of mental health inequities and support needs among Muslim communities.

## 2 Method

### 2.1 Consultation—An Approach

Consultations, a method of encouraging public and patient involvement and engagement (PPIE), are beneficial to identifying awareness and educational needs of future research topics and are further informed by those in need [33]. We adopted this method due to successful use when designing better healthcare standards and policies [34] as it presents opportunities to safely exchange experiences, opinions and perceptions between professionals and members of the public to improve healthcare delivery [35]. Consultees were invaluable in their willingness to provide insight into nuanced challenges to help identify areas for improvement based on their vast experiences.

### 2.2 Ethics

Ethical approval was not required for this PPIE consultation exercise. We did not involve the collection of data from consultees in ways that impacted their rights or welfare. Instead, we emphasised collaboration and consultation, which are essential aspects of the research design and implementation process, rather than conducting interventions or observations that might pose potential risks to consultees. We drew inspiration from previous studies and following our consultations, ethics approval was granted by the Research Ethics Panel of the School of Nursing and Advanced Practice from Liverpool John Moores University: NAH(PGT)3023 to further explore key findings from this consultation.

### 2.3 Theoretical Framework and Researchers' Positionality

The philosophy that underpinned our approach is that the phenomenon of equity in mental health care and support is rooted in subjectivist epistemological and relativist ontological foundations, that is, social constructivism [36]. With the context of our positionality as authors, researchers questioned their own assumptions as we played an integral role in data collection and interpretation [37]. Considering this, A.T., a British Palestinian Muslim, brought children's mental health nursing, perspectives on service delivery for the Muslim population, professional and personal perspectives from within the Muslim community in the Liverpool area. O.H. belongs to a minoritised ethnic community (MEC) in the United Kingdom and brought perspectives from working history as a nursing assistant caring for individuals with complex mental health conditions and lived experiences as a family carer. R.F. is a British Black Muslim with a keen interest in reducing health inequalities. S.D. is a Roman Catholic Christian, mixed race (White/Black Caribbean), mental health nurse with a special interest in culture within mental health services. H.I. is a Muslim who belongs to MEC in the United Kingdom with an interest in using participatory approaches in research and exploring barriers to seeking mental health support. Z.H.T. is a Muslim who belongs to MEC in the United Kingdom who brought children's nursing, specialist community health nursing, health visiting and perspectives of diversity and inclusion. C.E. identifies as White with no religion, a psychologist with expertise in mental health, public and patient involvement in research. P.S. is Hindu who belongs to a MEC in the United Kingdom, and is a Chartered Psychologist with expertise in health service usage, developing community‐based mental health interventions and implementation science. O.B. is Christian who belongs to a MEC in the United Kingdom and a mental health nurse with expertise in global mental health and service delivery. Our identities and previous knowledge afforded us opportunities to interrogate various interpretations of consultees' experiences.

### 2.4 Procedure

The core team met regularly to identify effective recruitment strategies and two members (A.T. and R.F.) led on recruitment due to having a shared background and pre‐existing connections to the communities of interest. Being conscious of the Muslim faith and customs, the team agreed to hold separate consultations for men and women in trusted locations through ongoing work within communities. Consultations were held at a multicultural community centre and a religion centre in Liverpool in June 2023. Consultees had to be aged 18 years and over.

A poster was created that included the purpose and focus of the consultations, the dates and location, what consultees should expect and an email address for registering interest. The invitational poster was shared to the specified communities by the recruitment leads and also sent by email via the host University to potential consultees. After receiving the invitations and contacting the consultation team to register their interest, consultees were provided with the time of the sessions to attend. We developed QR codes used to fill consent to contact for future studies and consultation feedback forms. £20 vouchers were offered to each participant to compensate for the time and knowledge shared.

### 2.5 Conducting the Consultations

Twenty‐seven consultees attended the women's consultation at a multicultural community centre and eight consultees attended the men's consultation at a religious centre. Consultees were individuals from Yemeni, Somali, Sudanese, Egyptian, Algerian, Pakistani and Moroccan communities who share the Islamic faith. The research team introduced themselves to consultees and a brief statement was made to remind consultees of the purpose of the consultation. Following this, Z.H.T. and A.T. facilitated the consultations and the following five key questions (informed by a scoping literature review exercise as well as experiences' of authors) were presented:

- What are your thoughts and opinions of mental health care and support in your community?
- What are your thoughts and opinions of mental health care and support provided from social care services and health services such as the NHS?
- What types of services (mental health care and support needs) would you like to see for your community?
- What would you like to see in the future from health and social care services?
- How do you see the relationship between your culture/beliefs and mental health/social care services?

**Figure 1:**
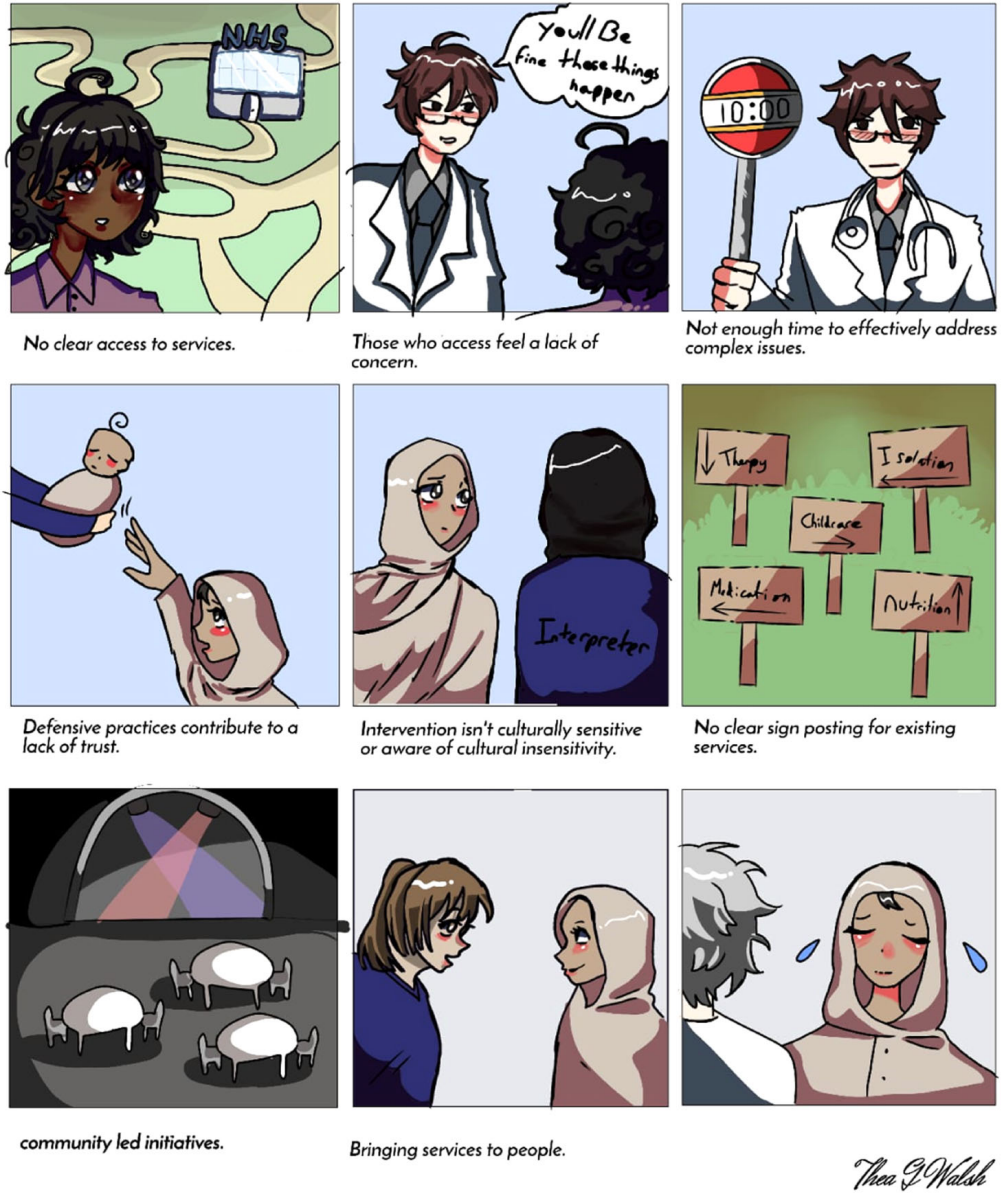
Factors impacting access to adequate mental health support amongst Muslims who belong to minortised ethnic communities in Liverpool (Women's group).

**Figure 2:**
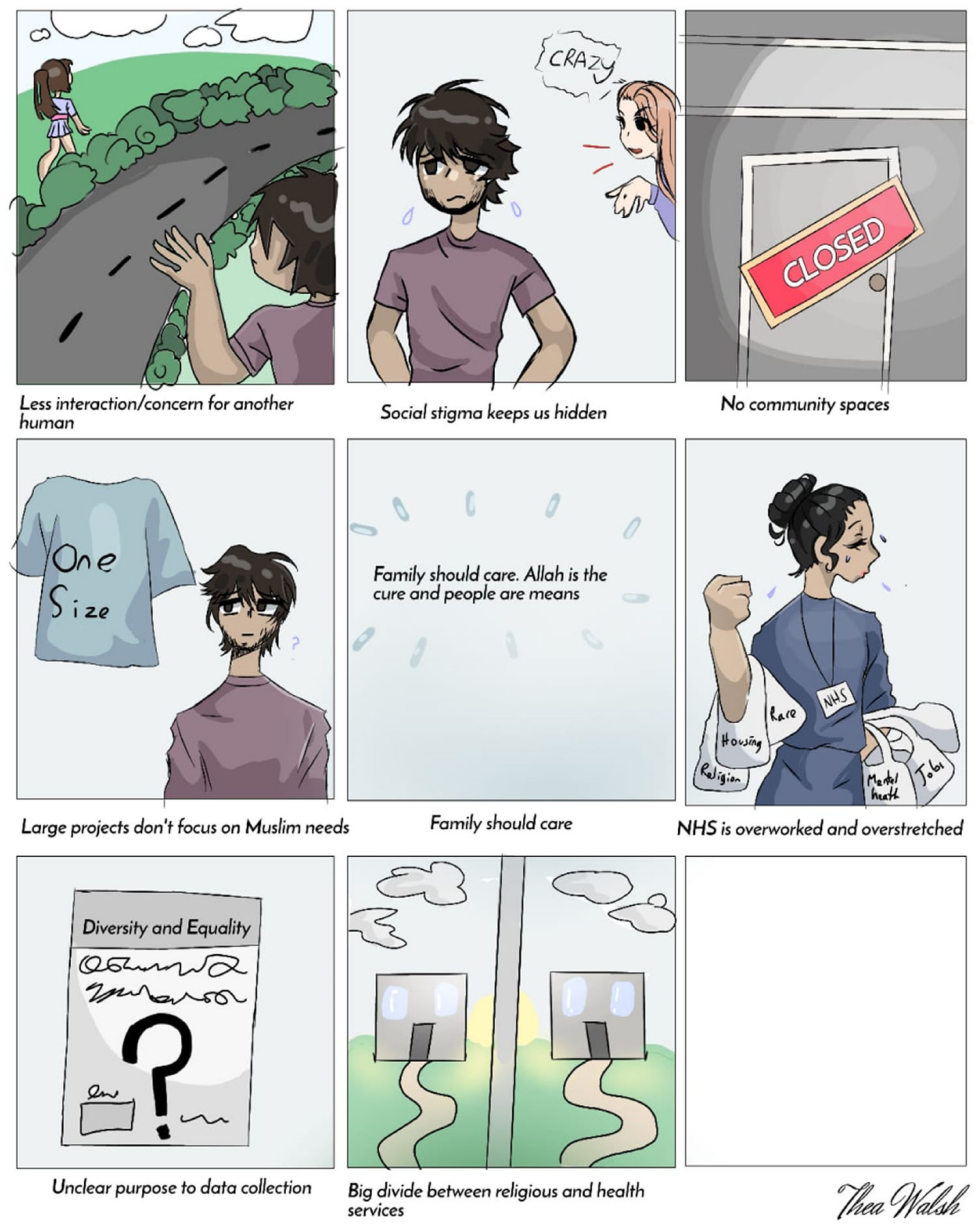
Factors impacting access to adequate mental health support amongst Muslims who belong to minoritised ethnic communities in Liverpool (Men's group).

Members of the team were responsible for taking notes/drawing during the sessions and watched out for any signs of distress amongst consultees. The purpose of the drawings was to create a visual representation of consultees' narratives that provides a snapshot of some of the main issues that were raised, which was shared with the consultees. The team followed the lead of consultees to allow for discussions to flow. Follow‐up questions were sometimes asked to provide clarity; otherwise, the discussions were allowed to happen naturally whilst the illustrator drew images that depicted some poignant and/or common experiences that the consultees shared (see Figures 1 and 2). We aim to use these drawings for exhibitions in the future. Consultations concluded with the team providing information about some local services that could be beneficial for people and exchanged contact details with some consultees who were actively advocating in the community for better mental health care and support. Consultations lasted for an average of 90 min. All consultees were able to speak English language.

### 2.6 Analytic Approach

Given the underlining research values and philosophical assumptions as established above within the theoretical framework and researchers' positionality section, reflexive thematic analysis (RTA) was the most appropriate for this project as it affords researchers the opportunity to interpret data and acknowledge their own influence on the analysis process [38]. RTA is also designed to be accessible for consultees–co‐researchers, particularly those new to research, such as A.T., O.H., S.D., R.F. and Z.H.T., and to facilitate their contribution to theme generation [39]. Given that meanings necessitate interpretations, we adopted semantic, deductive and inductive orientations [40, 41]. Being culturally aware, research team members who are Muslims advised not to record consultation sessions to allow consultees to talk without hesitation. During consultations, O.B., O.H. and S.D. took notes of consultees' narratives (transcripts). Transcripts were analysed thematically, following the six steps outlined by Braun and Clarke [40].

1. A.T., O.H., S.D. and O.B. initiated the analysis process by *familiarising* themselves with the transcripts.
2. They began *coding* by identifying segments of data that appeared to be potentially significant.
3. Individually, they proceeded to *generate initial themes* to identify shared patterns and meanings across the data set.
4. To ensure the viability of the overall analysis, A.T., O.H., S.D., O.B., R.F., Z.H.T. and H.I. *developed and reviewed themes* by discussing the relationships between them, ensuring that each theme conveyed a compelling narrative.
5. In the process of *refining, defining and naming themes*, C.E. and P.S. reviewed the analysis and suggested ways to fine‐tune the themes generated. These themes were also presented to groups who participated in the consultations and identified religious leaders for critical feedback to aid trustworthiness [42].
6. Critical feedback was taken into consideration during the writing‐up phase, to which all authors contributed.

### 2.7 Findings

Four key themes were identified from consultees' narratives and are presented in Table 1 below. It is relevant to note that these themes are inextricably linked and there are some overlaps in the information presented. However, each theme has its distinct focus, which are highlighted in the subthemes.

**Table 1:** Overview of generated themes.

Themes	Subthemes
Broken cycle of trust	Defensive practices
	Lack of safety and deepened stigma
	Quality of support
Overmedicalised model of care	Predominant emphasis on medication
	Default treatment incompatible with the culture and beliefs of consultees
	Exclusion or minimisation of holistic interventions
Community mental health prevention initiatives	Community network initiatives as a long‐term solution
	Limitations of formal services
	Bringing services to the community
Culturally conscious training and education	Nuanced training around religious and cultural complexities
	Improving mental health awareness and education in Muslim communities
	Bridging the gap between community initiatives and the NHS

### 2.8 Broken Cycle of Trust

#### 2.8.1 Defensive Practices

Concerning mental health care and accessing support for themselves and their loved ones, most consultees, especially women, either had more negative experiences or the incidents that they shared were significant enough to deter them from attempting to access services in the future. A shared consensus from both consultations was that they would not receive the right support through the NHS, and this was attributed to statutory services not having anything in place to help the Muslim community with mental health needs until they are in crisis. Even in crisis, some consultees shared how friends and family were failed by the system and turned away because healthcare professionals did not consider their conditions to be severe enough. Echoing this voice, most consultees believed that racism or discrimination is evident within mental health services, with healthcare professionals acting uncaringly and treating them differently by undermining their lived experiences, dismissing mental health as an illness and not following up with service users.

#### 2.8.2 Lack of Safety and Deepened Stigma

Another significant factor that deterred consultees from engaging with formal services was the unending fear of losing children to social services, having witnessed or heard of similar outcomes. Some consultees believed that social services were informed disproportionately when mental health support was sought by ethnic minorities. For example, a participant shared how an individual who was reading the bible as a coping strategy was reported to PREVENT as a risk of radicalisation. Such incidents have instilled concerns within the Muslim community. Although many consultees echoed the belief that mental health struggles should not be hidden, they acknowledged that feelings of shame were often attached, contributing further to stigma and lack of safety in seeking mental health support openly within the community or through statutory services. Despite recognising their resilience as a community, consultees believed that more positive narratives about mental health ought to be presented in their community.

#### 2.8.3 Quality of Support

Consultees reported that negative experiences proliferate faster within the community; a bad experience with a service would often led to majority of the community avoiding that service and also discouraging others. Overall, consultees stated in various ways how education and training surrounding culture, religion and mental health are needed for NHS service providers in reaching the Muslim ethnic minority community and for mending the severed trust. For instance, some explained how miscommunication, due to language inaccurately translated from the service user to the practitioner due to varying dialects from different regions, grossly impact diagnosis and strongly stated that something needs to be done about negligence. One participant spoke about how her effort at getting her mother better treatment made her act as a translator, but her limited grasp on local dialect meant that some nuance were lost during the process of communication with medical practitioners, which resulted in misdiagnosis. She spoke extensively about how she internalised the guilt and shame over a long period of time.

### 2.9 Overmedicalised Model of Care

#### 2.9.1 Predominant Emphasis on Medication

Current mental health care systems were reported to be too reliant on standardised medical approaches. Some consultees voiced that the NHS's approach ‘work at fixing the issues by painting over it [rather] than working on the different causes’. Consultees acknowledged that the NHS staff are overworked and stretched due to a broken system; therefore, struggling to solve complex problems and the idea of prescribing medication was described as ‘convenient’ for practitioners. Most believed that they are prescribed medicines partly due to a language barrier that led to misunderstanding and that older relatives (aged 60+) struggled to receive help due to this barrier. For most, practitioners place greater emphasis on pharmaceutical interventions when people attend their medical appointments. Consequently, most attributed this overmedicalised approach to politics around funding and shared their worries that medication is being pushed as a means of profiting rather than benefiting the population being served, which they felt has adverse side effects on people. One of the contributory factors for excess medication prescription raised by consultees was that General Practitioners (GPs) do not have time to listen to people anymore.

#### 2.9.2 Default Treatment Incompatible With the Culture and Beliefs of Consultees

A few consultees voiced that the NHS needed to recognise other options i.e., traditional, complementary and intergrated medicine as equivalent to medications and labelled them as an ‘alternative’ which currently carries negative connotations within the community, which suggests that these methods are secondary or inferior. Consultees shared that medical‐only approaches or people being sectioned under the Mental Health Act fails to acknowledge the roles of families within collectivist cultures, who are often not carried along. These experiences led to consultees' belief that doctors make assumptions and draw conclusions that may not reflect a person's needs adequately, for example, making stereotypical assumptions that someone from an ethnic minority background has lots of children and this must be the cause of their stress or depression rather than being clinically assessed adequately. Most consultees reported that some NHS mental health services are believed to be outdated in some approaches. They stated that appointments felt less personal, and the motivation observed was to get service‐users in and out without genuine and detailed consultations. Hence, most consultees opined that these default pathways are not culturally appropriate.

#### 2.9.3 Exclusion or Minimisation of Holistic Interventions

There was a consensus that indigenous white ethnic groups have branded holistic “bio‐psychosocial” interventions for themselves despite holistic interventions originating from third world countries, and ethnic minorities are being excluded from receiving these interventions. Consequently, a few consultees stated that they felt unsafe at the GP and left appointments more depressed than when they go in. Therefore, consultees believe that practitioners need to be educated on addressing issues from the root cause(s), and services need to offer holistic support.

### 2.10 Community Mental Health Prevention Initiatives

#### 2.10.1 Bringing Services to the Community

The majority believed that mental health services need to be brought to the community and more measures put in place to support minoritised ethnic communities in accessing mental health services, making room for understanding nuances in background and cultural differences, as a one size fits all approach does not work. Consultees largely agreed that most practitioners are culturally unaware themselves in ways that are unprofessional and potentially detrimental to well‐being of service‐users and patient. For instance, having a doctor who is an ethnic minority does not solve the issues resulting from a lack of diversity because if a doctor's culture and beliefs do not align with service users' culture, this can often lead to miscommunications and certain issues are ignored.

#### 2.10.2 Community Network Initiatives as a Long‐Term Solution

Recognising the limitations of formal services, most consultees repeatedly highlighted the benefits of a supportive community and its potential in meeting people's needs and merging the gaps that NHS services cannot fill. Generally, consultees' view that meaningful community support services should be reinstated and community delivery should be the default standard for mental health services. Most consultees shared their disappointment regarding the underfunding of local services and a few consultees suggested for the NHS to help out in the community by signposting service users and carers to the right people, making use of holistic practitioners, local organisations, charities, businesses and individuals who work in the field. For instance, consultees suggested the need for having weekly talks/small workshops that people can attend easily to discover services in their immediate vicinity. Many consultees believed that this is the only way the NHS can lessen current economic burden, by helping to rebuild and give back into the community to bridge existing treatment gap with regards to mental health care and support. Therefore, there needs to be an increase in community spaces like the Mosque, where people can talk about their mental health, have informal catch‐ups with mental health practitioners and group activities for people to share resources and enjoy one another's company.

#### 2.10.3 Limitations of Formal Services

The issue of limited time to conduct adequate clinical assessments, coupled with the culture clash and language barrier invoked stress for people affected by mental health difficulties. According to consultees', the NHS system is not prepared to address comorbid issues as they can only deal with one health challenge at a time. Another factor raised at both consultations is how generational trauma that many patients and their loved ones face as ethnic minorities in England are unacknowledged on a systemic level and the NHS is not prepared to deal with those matters. Therefore, a suggestion that many agreed with was that mental health should not be viewed solely through the medical lens. More specifically, the notion was that mental health should be dealt with as a separate service, as a consultee expressed, ‘doctors originally only healed cuts and scrapes’; therefore, setting up community projects would be a more beneficial and impactful preventative measure for the community before individuals reach a point of crisis that needs medical intervention. Consultees reported that doctors are restricted from providing useful information because of policies that prevent them from sharing holistic knowledge. It is believed that this would not be the case in a community space where doctors play a supporting role to community services.

### 2.11 Culturally Conscious Training and Education

#### 2.11.1 Nuanced Training Around Religious and Cultural Complexities

All consultees valued faith as it relates to healing, and they found the Mosque helpful in that regard. Many varied in their perception about the relationship between mental health and Islam, as some shared that Islam does recognise that it is an illness and not ‘black magic’ or the ‘evil eye’. Nevertheless, consultees agreed that there is cultural stigma surrounding openly admitting to mental health struggles. Consultees shared that many in the community are from countries where they must hide mental health struggles to avoid being labelled ‘mad’. A consultee shared that ‘there are more facilities here (UK) that offer mental health support than back home’; however, the majority of consultees shared that existing statutory services do not adequately reflect and encompass the needs of the Muslim community. There was consensus that these services fail to uphold their traditional values and added to existing stigma surrounding mental health and Islamophobia. Therefore, education and training were identified as a multifaceted need for practitioners to develop better understanding.

#### 2.11.2 Improving Mental Health Awareness and Education in Muslim Communities

Consultees suggested that there is a need for regular events and interaction with members of the community in their own setting to educate people about mental health conditions. This would increase people's awareness and understanding of general mental health and specific conditions, as it might relate to their circumstances, informing one another of services within their community that can support them whilst being conscious of their cultural needs. Few believed that this would be useful in the journey of destigmatising mental health. While some believed that the NHS system needed to be changed completely, others stated that it was not necessary to disband the system, but that improvements and customisations to individual needs are paramount.

#### 2.11.3 Bridging the Gap Between Community Initiatives and the NHS

Consultees shared that the GP should know the cultural background of their patient so that they can accommodate for this and tailor the care experience better to include a person's cultural needs. Practitioners need to listen to the patient better and more confidence needs to be nurtured between people and the GPs so that they feel empowered to disclose information concerning mental health challenges without fearing extreme repercussions. Consultees also felt that staff turnover was too high, which contributed to the lack of confidence in the service. Hence, more trust needs to be created through investment in the community, education and training that is conscious about the nuance and complexity of how mental health interacts with ethnicity, religion and other life circumstances.

Visuals depicting consultees' narratives from both consultations are presented in Figures 1 and 2.

## 3 Discussion

Our consultations aimed to better understand mental health needs and support of minoritised ethnic Muslim communities in Liverpool by exploring mental health inequities. To our knowledge, this is the first study to adopt a constructivist paradigm through consultation, which resonates with key principles of creative qualitative research methods to enable knowledge generation and exchange within minoritised ethnic communities as it pertains to mental health inequities. There remains a dearth of evidence around the concept of communities of practice in relation to mental health, which encompasses bringing people with shared interests together through active learning to engage on relevant practices, underpinned by relevant methods or tools for learning [43]. Addressing this knowledge gap has implications for building mental health research capacity, as it showcases the benefit of socialised power. Four themes were generated from our consultations, reflecting experiences of multiple barriers to engagement with mental health services. Central across these themes is the impact of culture and the need to understand cultural nuances to engage people better as advocates in their care. Considering that the NHS is founded on principles of equality and social justice, it is crucial for NHS organisations to consciously embrace inclusive language and approaches needed to play an active role in becoming ‘unapologetically anti‐racist’ [44].

A key consideration is the biomedical model of mental health [45], whereby the medical profession is positioned as holding knowledge and power, and patients seen as needing their help. Much has been written in the literature about the shift in positionality and power as the recovery movement emerged [46]. As service‐user involvement in mental health care has developed, better partnership working between professionals and service users and a rejection of the medical model are advocated in the literature [47]. However, monocultural western European–American healthcare models of patient–doctor communication remain dominant [48]. This has implications both in terms of power balances and also how symptoms are expressed and recognised. Culturally, specific constructs of illness can inform how individuals perceive (and therefore present) their symptoms (e.g., physical/somatic rather than mental health related) [49]. Such instances are reflected in our consultation discussion, where concern regarding the overmedicalisation of mental health care, rather than holistic packages, was highlighted. Other than language, cultural differences are largely unexplored. Even when language is considered, our consultees highlighted negligence relating to translations and dialects, which can lead to miscommunication and misdiagnosis.

Additionally, findings from our consultations suggest that having more Muslim practitioners for representation purposes could improve psychological safety and promote better engagement with formal services. However, Muslims sometimes are hesitant to be treated or assessed for mental health‐related issues by someone from the same ethnic background due to confidentiality being breached, that is, that they may have links with their social circle and divulge sensitive information [50]. In support of key findings from our consultations, emerging evidence suggests that NHS organisations are still at a stage of infancy in terms of providing adequate and responsive feedback [51, 52, 53]. This has implications for patient safety and ultimately, service users' and carers' satisfaction, especially within Muslim communities, where there remain a lot of misconceptions relating to mental health. In the absence of adequate and timely support, individuals within these communities are likely to put more pressure on themselves and aggravate their difficulties. Despite increasing need for mental health support globally, a very small proportion of people receive minimal and adequate treatment [54]. Therefore, to improve public mental health needs, there is an urgent need to deepen the understanding of the populations being served through meaningful consultations aimed at amending existing flaws within statutory services that breach the right to health [54].

Within the context of our findings, there is an urgent need to rethink processes involved in mental health needs assessment for people who are Muslims from minoritised ethnic communities. Also, there is an urgent need to reimagine mental health services' research capability building by placing more emphasis on collaborative advocacy and adopting place‐based and integrative approaches [54]. Our discussions highlight continued epistemic injustice in mental health care among Muslim populations in Liverpool, UK. Whilst there have been improvements overall in terms of lived experience involvement in recognising the epistemic power dynamics within mental health care [55], our consultations highlight that not enough effort is being made to reach these populations by researchers, policy makers, or health professionals, despite continued known health inequalities. Accessing appropriate mental health services was well articulated to be a significant problem as consultees acknowledged that they are unaware of early intervention services that are tailored for the diverse Muslim community in deprived areas of Liverpool, despite demonstrating autonomy in their rights to being involved in their health care choices.

Minoritised ethnic communities are still under‐represented in the United Kingdom psychological services such as Improving Access to Psychological Service (IAPT) [56], and this populations' clinical outcomes can also be poor [57]. A Positive Practice Guide was developed to achieve access and outcome equity for minoritised ethnic communities [56]. The Guide was co‐developed by IAPT clinicians and minoritised ethnic service users. Separate sections cover increasing access, reducing non‐attendance rates, developing an appropriately skilled workforce, and ensuring that the core principles of effective psychological therapies are delivered in a culturally sensitive manner. Process research evaluations are needed within organisations implementing psychological interventions tailored with minoritised ethnic communities to ensure service users' needs are being met to mitigate existing health inequalities, which is on the increase. Working alongside communities not only in considering mental health literacy [58], but also supporting practitioners in culturally conscious training to support shared decision making about care options is recommended.

There is a need to co‐produce participant information to serve the population, but also to provide accurate and timely interpretation at health care appointments [59]. Organisations have a responsibility to deliver culturally informed care. First, service providers and researchers need to understand the diversity within ethnic minority groups [60]. Second, cultural differences should be better understood. Third, understanding generational trauma [61] and historic racism [15] to support staff in understanding how services are perceived, giving voice to concerns about referrals to other support services and how to navigate these. In this community engagement paper, specific actions to improve uptake of mental health services include community‐based interventions including (1) training and education as it pertains to intersectionality, that is, multiple identities, which shapes peoples' experiences, (2) social prescription and accessibility, (3) representation as it pertains to being racially inclusive during engagement/consultation.

Given that one of the four key aims of Integrated Care Systems is to address inequalities in health outcomes, experience and access [62], it is crucial to have consultations set out to accommodate diverse stakeholders and especially those from minoritised ethnic communities with an overarching aim of deepening understanding of mental health support needs. Such processes would afford staff more opportunities to learn first‐hand about racism and or islamophobia as it applies to health care provision. It may also provide staff opportunities to learn or adopt new approaches to advocate for service users and carers' personal recovery. Given the dearth of social prescription amongst communities with high numbers of people from minoritised ethnic groups, consultations based at familiar local environments such as religious or cultural centres as an outreach may help with access [63].

Consultations as a method of engagement with peoples' lived experiences would be the most appropriate approach to address workforce knowledge or manage populations' expectations and it is crucial to ensure that community engagement is racially inclusive and well‐resourced in terms of time, expertise, and budget [64]. Further, such avenues would present opportunities to better operationalise key anti‐racist principles [44], through active engagement of minoritised ethnic communities in various research activities to understand lived experiences, strengthen partnerships and collaborations aimed at supporting delivery of evidence based mental health care provisions to tackle inequalities. We urgently need future mental health research priority setting with minoritised groups, that is underpinned by participatory action research design, to simultaneously examine and proffer evidence‐based recommendations to addressing mental health inequities, as it pertains to Muslims living in deprived areas.

### 3.1 Strengths and Challenges Associated With Consultation

Public consultations play a role in service development. Quantitative approaches tend to be a very common approach adopted for public consultations as it can be relatively easy to refer to within statutory reports. Our consultation adopted a qualitative approach to provide data for future research work relating to mental health inequities within Liverpool City Region, but equally a way to build trust within deprived communities. A key strength for this consultation was how quick we were able to recruit people from diverse communities for our consultations, primarily due to concerted effort led by R.F., a local councillor within the area. Positive feedback received after the consultations mostly had to do with our appropriate approach and dialogue methods as we largely allowed consultees to take control of what was being said. More importantly, they expressed how comfortable they felt within centres of choice (multicultural community and religious centre). Overall, consultees shared during consultations that they experienced the cathartic effect of talking through difficult topics with validation from other consultees over food and drinks of their choice. Contrary to the notion that minoritised ethnic communities are ‘hard to reach’ [65, 66], our consultations suggest that these groups do speak out loud and clear, yet remain seldomly heard. The positive approach to listening and responding to the experiences of people in different communities is advocated as a method to enrich discourses. Whilst being led by consultees in not recording the sessions to allow for free discussions—an approach considered to be culturally sensitive, this did limit the researchers' ability for verbatim transcription. Although connections could be established at religious and cultural centres, we acknowledge that men's consultation session did not yield much interest. As such, an alternative approach could have involved reaching out to local sports teams, for instance.

Even though all consultees are Muslims, our work could have been strengthened by linking up what was said with their ethnicity, which would have provided us with more understanding of what some groups of Muslims find useful in terms of existing services compared to other groups of Muslims within the consultations. Whilst consultation is a legitimate step in public participation, we acknowledge it remains low in terms of citizen power [67]. In line with the Organisation for Economic Co‐operation and Development (OECD) active participation framework, we view our consultations as a two way relationship, which can be used as means to provide feedback to services [33] and can be used for future mental health research priority setting.

## 4 Conclusion

Muslims from minoritised ethnic communities living in deprived areas in Liverpool face significant barriers to accessing statutory mental health services. Improving their access to mental health services requires practical support like offering culturally and religiously appropriate information and improving local channels of communication (e.g., through faith leaders). Setting up consultations with diverse stakeholders in mental health within local communities can increase awareness concerning common challenges people face, offer reassurance and validation. Consultations can also help normalise conversations about mental health and ultimately, the process of building trust needed for building research capacity, facilitating meaningful co‐production and service re‐development. Whilst consultations can accommodate existing mental health provisions within statutory services, which would be relevant to some people, it is important for Integrated Care Systems to financially invest in place‐based partnerships to encourage regular consultations, potentially attracting more stakeholders needed to tackle mental health inequities and offer timely interventions. Equally, consultations could be an avenue for stakeholders, such as mental health practitioners, to learn from minoritised ethnic communities directly to help mitigate existing animosity amongst stakeholders and support the ambition of existing anti‐racist framework.

## Author Contributions

**Ashraf Tannerah**: conceptualisation, writing–original draft, writing–review and editing, formal analysis, project administration, validation, resources. **Oluwalolami Hazel**: writing–original draft, writing–review and editing, formal analysis, visualisation. **Sheree Desson**: writing–review and editing, formal analysis, resources. **Rahima Farah**: formal analysis, resources, validation. **Zalihe Kamil‐Thomas**: writing–review and editing, formal analysis, supervision, funding acquisition. **Halima Iqbal**: writing–review and editing. **Catrin Eames**: methodology, writing–review and editing, writing–original draft. **Pooja Saini**: methodology, writing–review and editing, writing–original draft. **Oladayo Bifarin**: funding acquisition, writing–original draft, writing–review and editing, conceptualisation, methodology, data curation, formal analysis, supervision, investigation, visualisation, resources.

## Conflicts of Interest

Oladayo Bifarin is a National Institute for Health and Care Research Leader. The views expressed in this article are those of the author(s) and not necessarily those of NIHR or the Department of Health and Social Care.

## Data Availability

Research data are not shared.
